# Lord Lister: VIII: Through Pus to Surgical Cleanliness

**Published:** 1918-03-16

**Authors:** 


### LORD LISTER.

### VIII.—Through Pus to Surgical Cleanliness.

The problems that Lister set himself to solve are now clear. He desired to cleanse an accidentally inflicted wound from all pathogenic germs that had settled upon it from the air or been introduced into it by contact with contaminated solid or liquid, and he desired to prevent any subsequent contamination of the wound thus cleansed. It was not for a year or two after he had applied this principle, which we now know as surgical cleanliness, to accidental wounds that he applied in practice the latter part of it to the wounds inflicted by the surgeon himself.

These are the principles of Listerism. These are the bases of the doctrine he taught, and to compass these ends he experimented with a very great variety of means, and was perpetually occupied in trying new antiseptics and in varying the details of his practice so as to surmount difficulties, of which every alteration of method introduced a new crop. Nevertheless, many of his contemporaries chose to consider, not only that he was wedded to carbolic acid as a germicide, but that his practice differed in no way from the established practice except in the employment of carbolic acid as a dressing; and there are still some persons so ignorant as to suppose that his concern was solely with the cleansing of accidentally inflicted wounds, and that he paid no regard to what we now call asepsis—that is to say, the prevention of contamination of those wounds that the surgeon himself inflicts. The two practices are so obviously and so equally corollaries of the one discovery that the disastrous course of wounds is due to contamination by living germs, that a man who should grasp the one and not the other must be an idiot; and Lister was not an idiot; but there are some who think, or at any rate say, that his theory and practice were thus limited.

For the purpose of cleansing an accidental wound that was known or presumed to be already contaminated, Lister employed carbolic acid in what we should now regard as the very excessive strength of one part of the acid to twenty of water—a saturated solution. If the wound was large, the raw surface was swabbed with lint dipped in this solution. If the orifice in the skin was small, such as that produced by the penetration from within of the pointed end of a fractured bone, the nozzle of a syringe was inserted into the orifice, which it plugged, and the interior cavity was distended with the solution of carbolic acid under pressure. So strong a caustic killed the tissue with which it came in contact, so that the surface sloughed, and suppuration, so far from being prevented, was necessitated. However, the primary purpose of the operation, the destruction of the pathogenic microbes, was usually effected, and such wounds usually, though not always, healed without much delay or much suppuration.

A necessary complement of destroying any microbes that might have gained access to an accidentally inflicted wound is the prevention of subsequent contamination, and this, the aseptic system proper, was the chief object of Lister's solicitude. The efforts must be directed to two chief objects that are in practice distinct: first, the prevention of contamination during operation, whether the operation is the cleansing of an accidental wound or the intentional infliction of a wound by the surgeon; and, second, the prevention of subsequent contamination.

Lister began with the notion then universally prevalent, and firmly fixed in his mind, that the chief source of contamination during operation was the air. There was good reason for this belief. Most of the experiments of Pasteur were founded upon it, and went to corroborate it. Pasteur's experiments were mainly directed to show that putrescible and fermentible matter remained sweet, unputrefied and unfermented, as long as the air that gained access to it was freed from living microbes. The air was allowed to have free access to the fermentible or putrescible matters, but it was shown conclusively that on the one hand, if the air was not filtered, putrefaction or fermentation inevitably took place; and on the other that, if the air was effectually filtered, the putrescible or fermentible matter could be preserved for an indefinite length of time without either of these changes taking place. And suppuration was inseparably connected in Lister's mind with putrefaction or "decomposition."

An immense corroboration of this belief in the paramount infective property of the air was effected by the researches of Tyndall into atmospheric dust. Tyndall was one of the two or three most prominent physicists of his day. He was a most skilful deviser of crucial experiments; he was a vigorous controversialist; he had a great gift of clear and convincing exposition; he wrote pure and trenchant English; and he had the ear of the public. Any cause that he advocated was secure of attention. Tyndall's experiments on dust in the air were most convincing. By passing a ray of brilliant light through a sample of air, the rest of the room being in obscurity, he showed up the floating motes of dust in the same way that they are shown in a slanting sunbeam, but far more conspicuously. Then he showed various methods of depriving the air of this dust, and proved incontestably that it could be deprived of them and rendered "optically pure." He showed that sterilised putrescible and fermentible substances exposed to this optically

☛ Previous articles appeared Jan. 26, Feb. 2, 9, 16, 23, March 2 and 9, p. 483.

**LORD LISTER**—(*continued*).

pure air remained sterile; and that when impure or ordinary air was admitted to them, organisms grew in them in great abundance. These experiments of Tyndall's attracted much attention at the time, and had, as Sir Rickman Godlee points out, a two-fold effect on Listerism, part good and part bad. They were good in as far as they rendered the germs, or what passed as the germs, actually visible, and thus confounded the sceptics who denied their existence; they were bad inasmuch as they tended to exaggerate still more the part played by the air in the contamination of wounds.

It was this rooted belief in the efficacy of the air as the chief and almost the sole contaminating agent that led many surgeons, though not Lister himself, to neglect the sterilisation of their hands and instruments, and it was the same belief that led Lister to introduce the spray as a seemingly necessary precaution in all cutting operations. Its use rested on the beliefs that the air was thick with pathogenic germs, and that the drops of carbolic-acid solution in the spray and the vapour they diffused would kill these germs instantaneously and render the air sterile. Neither belief was well founded, but the incorrectness of the one counteracted the incorrectness of the other. The germs would not be instantaneously killed by the spray; but then there are very few pathogenic germs in the air to kill. The motes that float so thick in every sunbeam are not all charged with germs, and the germs with which many of them are charged are not all pathogenic; and the few pathogenic germs that there are may, as they fall singly or a few at a time on to the raw surface of the wound, be safely left to be disposed of by the phagocyte cells that are awaiting them there. The spray, therefore, is practically useless, and, after continuous use for ten years, it was at length abandoned as the true facts were discovered. At long length it was realised that the important infecting agent is not the air, but the surgeon; and that the most necessary precaution to take is to sterilise not the air, but the surgeon's instruments, appliances, and hands. It was because he was so extremely careful of these, among other precautions, that Lister's practice was so successful. It was because, in consequence of Lister's teaching, they attended to these precautions, that other surgeons, who repudiated Lister's theory and his practice in other respects, obtained nevertheless some excellent results. These results, which should have redounded to Lister's credit, and should have gone to reinforce his teaching, were quoted against him, as showing that his methods were unnecessary. They were, in fact, due to the adoption of the vital part of his method.

The after care of wounds subsequent to operation was to be governed, according to Lister, on the same principle of preventing the access of pathogenic germs to the wound. This was attended by two special difficulties: first, the escape of the discharge, which in all wounds treated before his day had been profuse, purulent, and fœtid; and second, the protrusion from the wound of the ligatures by which the arteries had been tied. These, composed as they were of flax, cotton, or silk, contained, of course, both on their surface and in their substance, an abundance of microbes; so that however perfect the sterilisation of the surfaces of the wound itself, such sterilisation would be futile if infection were introduced by the ligatures. Obviously, it was necessary to sterilise the ligatures also; and this Lister recognised, and this he did. But the ligatures, even if sterilised, had to "come away"; that is to say, they must slough through the artery round which they were tied, and their ends must be left long enough to protrude from the wound, so that when the sloughing was complete the protruding end could be drawn upon, and the whole ligature gently removed from the wound.

In these two circumstances lay many difficulties that were counteracted by various expedients. The adjacent surfaces of the wound could not adhere together and heal as long as they were kept apart by a quantity of discharge, blood, serum, and pus, that filled the cavity of the wound as the water in a rubber hot-water bottle fills the bottle and keeps its sides apart. This difficulty was comparatively easy to overcome. In the first place, Lister realised the importance of a dry wound. The practice had been to tie every artery that spouted, but to ignore the oozing from smaller vessels, which continued to ooze when the wound was closed, and at length produced a considerable accumulation of blood in the cavity. Lister not only tied the spouting arteries, but stopped the oozing from the smaller vessels also, and thus dried up one source of the discharge. The other most unexpectedly disappeared of its own accord. As soon as the infection of wounds ceased, and the raw surface was no longer cauterised by strong carbolic solutions, the copious foul-smelling pus was no longer generated. It is true that this was what Lister was striving for and hoped for, but when it actually occurred, it gave him almost as much surprise as satisfaction. It was a marvel. It was incredible; and by those who had not actually witnessed the result in his practice it was long before it was believed. It was some time also before the result was actually attained.

(*To be continued.*)

